# The long-term psychosocial consequences of screen-detected ductal carcinoma in situ and invasive breast cancer

**DOI:** 10.1016/j.breast.2023.06.003

**Published:** 2023-06-07

**Authors:** Emma Grundtvig Gram, Túlia Filipa Roberto Manso, Bruno Heleno, Volkert Siersma, Jessica á Rogvi, John Brandt Brodersen

**Affiliations:** a Center for General Practice, Department of Public Health, University of Copenhagen, Copenhagen, Denmark; b Primary Health Care Research Unit, Region Zealand, Denmark; c Department of Geriatric and Palliative Medicine, Bispebjerg and Frederiksberg Hospital, The Capital Region of Denmark, Denmark; d CHRC, NOVA Medical School, Faculdade de Ciências Médicas, NMS, FCM, Universidade Nova de Lisboa, Lisboa, Portugal; e Research Unit for General Practice, Department of Community Medicine, Faculty of Health Sciences, UiT the Arctic University of Norway, Tromsø, Norway

**Keywords:** Screening mammography, Psychosocial consequences, Ductal carcinoma in situ, Invasive breast cancer, Nomenclature, Overtreatment

## Abstract

**Objective:**

Ductal carcinoma in situ (DCIS) is a risk factor for invasive breast cancer (IBC). The prognosis of DCIS is considerably better than for IBC, yet women do not distinguish between the threat. We aimed to compare the psychosocial consequences of screen-detected DCIS and IBC, and to examine this comparison over time.

**Methods:**

We surveyed a Danish mammography-screening cohort from 2004 to 2018. We assessed outcomes at six-time points: baseline, 1, 6, 18, 36 months, and 14 years after the screening. We measured psychosocial consequences with the Consequences Of Screening – Breast Cancer (COS-BC): a condition-specific questionnaire that is psychometrically validated and encompasses 14 psychosocial dimensions. We used weighted linear models with generalized estimating equations to compare responses between groups. We used a 1% level of significance.

**Results:**

170 out of 1309 women were diagnosed with breast cancer (13.0%). 23 were diagnosed with DCIS (13.5%) and 147 with IBC (86.5%). From baseline to six months after diagnosis, there were no significant differences between women with DCIS and IBC. However, mean scores indicated that IBC generally was more affected than DCIS. After six months, we observed that women with DCIS and IBC might be affected differently in the long term; mean scores and mean differences showed that IBC were more affected on some scales, while DCIS were on others.

**Conclusion:**

Overall, the DCIS and IBC experienced similar levels of psychosocial consequences. Women might benefit from renaming DCIS to exclude cancer nomenclature.

## Highlights

• Many women are not aware of the conceptual and prognostic distinctions between DCIS and IBC.
• In the first six months, there were no psychosocial differences between DCIS and IBC.
• Over time, there was a non-significant trend of worse psychosocial outcomes in women with IBC.
• Renaming DCIS to exclude cancer nomenclature may lower the psychosocial consequences of DCIS.

## 1 Introduction

Ductal carcinoma in situ (DCIS) is a localized cancer of the breast epithelia located in the ductal glands. As opposed to invasive breast cancer (IBC), DCIS has not spread beyond the ductal gland. DCIS is a risk factor for IBC; about 20% of DCIS progresses into IBC [1,2]. As with other cancer diseases, localized carcinomas have a better prognosis compared to invasive cancers. The risk of dying from breast cancer 20 years after a diagnosis of DCIS is about 3.5%, which is twice the rate of the background population [1,[3], [4], [5], [6]]. In contrast, the risk of dying from an IBC diagnosis is about 30% 20 years after. In other words, the risk of dying from IBC is about ten times as high as for DCIS [[7], [8], [9]].

Cases of DCIS account for 15–25% of screen-detected breast cancers [10]. Breast cancer screening aims to identify breast cancer at a localized stage to secure more lenient treatment and a better prognosis. Most DCIS are found through screening as DCIS does not cause symptoms before it becomes invasive. However, the biological nature of DCIS dictates that only a few cases of DCIS will go on to cause symptoms, progress to invasive cancer, or cause death [1,2]. Therefore, most DCIS cases detected at screening will be overdiagnosed, potentially leading to overtreatment [[11], [12], [13], [14]]. This is because we do not have any reliable prognostic markers, technologies, or diagnostic tools that can predict the lethal potential of the DCIS; to tell us whether the DCIS will progress to IBC or not. Before the implementation of breast cancer screening, DCIS was a rare diagnosis. Due to the growing use of screening mammography, the incidence has increased dramatically [12,15].

Being diagnosed with breast cancer, whether it is DCIS or IBC, has several short- and long-term consequences. Aside from the seeming health threat, women face life-long surveillance and side effects from the treatment. Despite the much better prognosis for women diagnosed with DCIS than for those with IBC, studies have indicated that women do not differentiate the health risk of DCIS and IBC. This can potentially result in unnecessary and exaggerated psychological distress in women diagnosed with DCIS [12,[15], [16], [17], [18], [19], [20], [21], [22], [23]].

The objective of this study is to compare the long-term psychosocial consequences of being diagnosed with DCIS compared to IBC in screening mammography and examine trends over time.

## 2 Methods

This is a cohort study with a 14-year follow-up which uses a condition-specific questionnaire. This study is a post-hoc analysis of a cohort study on the long-term psychosocial consequences of false-positive screening mammography [24,25]. The cohort consists of women who had breast cancer diagnosed by screening in 2004–2005 in Denmark. At inception, each of these was matched with women who had normal mammograms. We have assessed the psychosocial consequences with the condition-specific questionnaire Consequences Of Screening – Breast Cancer (COS-BC). We assessed the outcome at six-time points: baseline, 1, 6, 18, and 36 months, and finally 14 years after diagnosis. Information about survey administration and sampling strategy is available in the reporting of the primary cohort study [24].

### 2.1 Outcome measure - COS-BC

The COS-BC is a condition-specific questionnaire developed and validated to measure the psychosocial consequences of screening mammography [26,27]. The content validity was tested in focus groups and individual interviews, which established relevance, coverage, and understandability. The psychosocial domains and items were confirmed as valid scales using Rasch analyses [26,27]. The COS-BC originally included 12 domains but was revised to include two additional scales, which were first added to the COS-BC at the 14-year follow-up; empathy and impulsivity [28]. Therefore, these two scales are only measured at the last follow-up [25].

COS-BC part I consists of eight scales on negative consequences: Sense of dejection, Anxiety, Breast examination, and Negative impact on sexuality, Behaviour and Sleep, and two single items: Keeping my mind off things and Feeling less attractive. These items are scored ‘Not at all’, ‘A bit’, ‘Quite a bit’ to ‘A lot’. Part II consists of six scales measuring changes in psychosocial aspects: Worries about cancer, Social network, Existential values, Inner calmness, Impulsivity, and Empathy. These items are scored ‘Much less’, ‘Less’, ‘Same as before’, ‘More’, and ‘Much more’. This scoring reflects ‘Same as before’ as the scale's midpoint and indicates no change. As part II reflects changes, it was not included in the baseline measurement. In both part I and II, higher scores indicate more psychosocial consequences. Items on sociodemographic information were included in the questionnaire at baseline.

### 2.2 Statistical analysis

We compared the distribution of sociodemographic covariates with a χ^2^ test to examine comparability and potential confounding.

We compared the means of each COS-BC scale between DCIS and IBC with weighted multivariable linear models. Analyses were adjusted for potential confounders: employment status, socioeconomic status, age (quadratic), and civil status. We used generalized estimating equations (GEE) to account for correlated and overdispersed data due to repeated measurements and weighting. We weighted the models using inverse probability weighting [29] which adjusts for potential bias due to differential attrition. We modelled the probability of being not missing with logistic regression using the baseline covariates, diagnosis, previous scores on the corresponding scale, and response at previous follow-ups as covariates, and then estimated weights by the inverse of this probability. We lowered the level of significance to 1% (*P*<0.01) to avoid type-1 errors due to multiple testing. All analyses were performed with SAS 9.4 (SAS Institute, Inc., Cary, NC).

Unadjusted analyses were used to plot the mean scores at the various follow-up time points for the two groups together with the mean scores for the normal group to be used as a benchmark for the level of psychosocial consequences.

## 3 Results

**Fig. 1:**
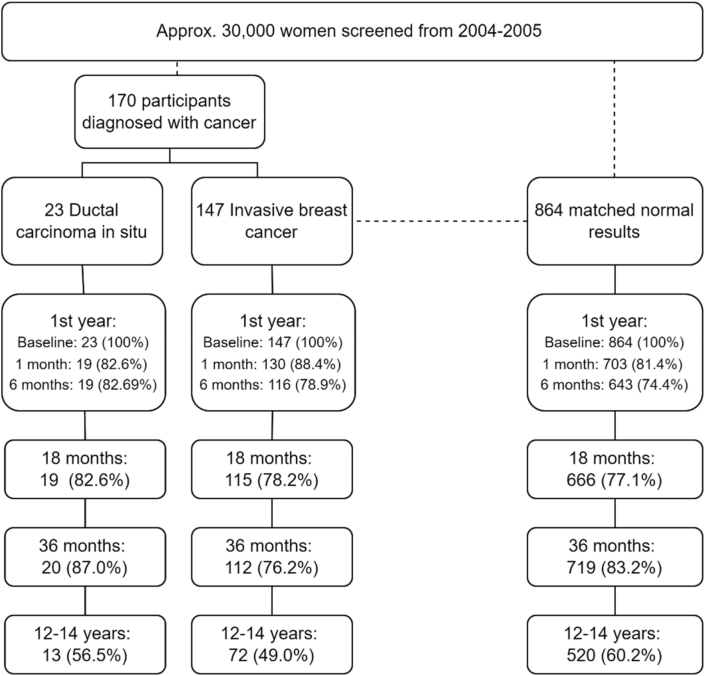
Flow chart and response rate.

About 30,000 women participated in mammography screening in the two geographical areas in the given period. Of all those, 230 women were diagnosed with breast cancer, either DCIS or IBC and 170 of those agreed to participate in our survey (74%). Of those agreeing to participate in the present survey, 23 were diagnosed with DCIS and 147 were diagnosed with IBC. The response rate at the 14-year follow-up was 56.5% and 49.0% for DCIS and IBC respectively (Fig. 1).

We found no significant differences between women with DCIS and women with IBC concerning age, social status, employment, and whether they lived alone (Table 1). As expected, the distribution of age differed slightly; women with IBC and women with DCIS were more likely to be older than women with normal results (Table 1).

**Table 1:** Study population baseline characteristics. ^a^p-value of a test of DCIS against invasive cancer. ^b^p-value of a test of all screening groups.

			Screening result			
		DCIS	IBC	Normal	Total	*χ2* p-value
		(n = 23)	(n = 147)	(n = 864)	(n = 1034)	
		**n (%)**	**n (%)**	**n (%)**	**n (%)**	
Age	50–54	3 (13.1)	33 (22.5)	217 (25.1)	253 (24.4)	0.61^a^
	55–59	7 (30.4)	31 (21.1)	310 (35.9)	348 (33.7)	<0.001^b^
	60–64	7 (30.4)	38 (25.9)	210 (24.3)	255 (24.7)	
	≥65	6 (26.1)	45 (30.5)	127 (14.7)	178 (17.2)	
	Missing	0	0	0	0	
Living alone (cohabitation)	No	20 (87.0)	102 (72.3)	611 (72.1)	733 (72.5)	0.20^a^
	Yes	3 (13.0)	39 (27.7)	236 (27.9)	278 (27.5)	0.29^b^
	Missing	0	6	17	23	
Employment	Working	6 (26.1)	73 (51.8)	467 (55.3)	546 (54.1)	0.046^a^
	Unemployed	0 (0.0)	5 (3.5)	38 (4.5)	43 (4.3)	0.06^b^
	Pensioned	17 (73.9)	63 (44.7)	340 (40.2)	420 (41.6)	
	Missing	0	6	19	25	
Socioeconomic Status	I	1 (4.4)	4 (2.8)	32 (3.8)	37 (3.7)	0.86^a^
	II	2 (8.7)	17 (12.1)	128 (15.1)	147 (14.5)	0.40^b^
	III	5 (21.7)	27 (19.1)	165 (19.5)	197 (19.5)	
	IV	9 (39.1)	43 (30.5)	312 (36.8)	364 (36.0)	
	V	6 (26.1)	50 (35.5)	210 (24.8)	266 (26.3)	
	Missing	0	6	17	23	

**Fig. 2:**
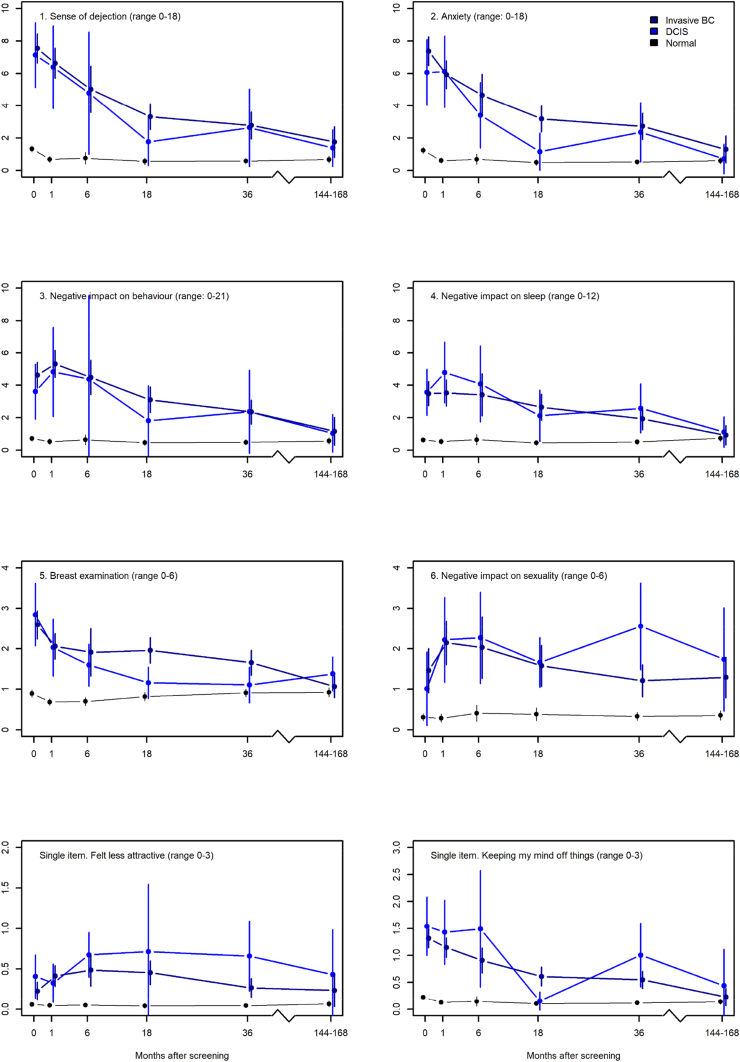
The mean scores and corresponding 99% confidence intervals of the COS-BC part I (y-axis) for the three screening groups at six time points: 0, 1, 6, 18, 36 months, and 14 years (x-axis). Higher scores indicate more psychosocial consequences.

**Fig. 3:**
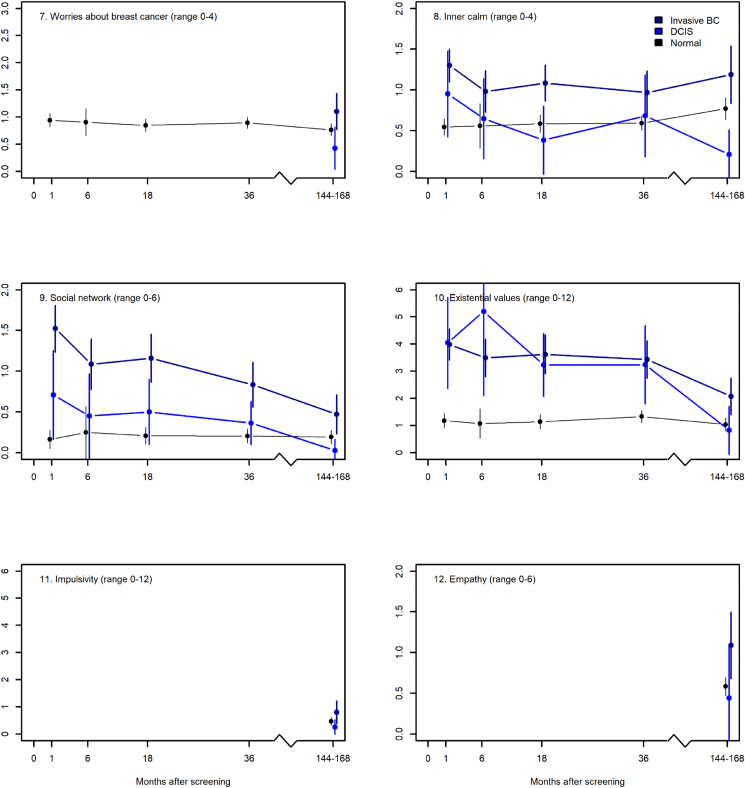
The mean scores and corresponding 99% confidence intervals of the COS-BC part II (y-axis) for the three screening groups at six time points: 0, 1, 6, 18, 36 months, and 14 years (x-axis). Higher scores indicate more psychosocial consequences.

Fig. 2, Fig. 3 show the mean scores of the two groups and for reference, it also presents data for women with normal results. In Fig. 2, IBC and DCIS generally have similar scores higher than those with normal results, which scores are stable over time and close to zero. In addition, the scores of DCIS and IBC approach the scores of normal results but do not overlap with those of normal results on the scale 6. At most times points, IBC has higher mean scores than DCIS on scale 1, 2, 3, and 5 and vice versa: DCIS has higher mean scores than IBC on scale 4, 6, 7, and 8 (Fig. 2). In all these cases the confidence intervals overlap.

On all scales in part II, IBC has higher mean scores than DCIS and normal results (Fig. 3). Over time, the scores approach those of normal results and confidence intervals overlap.

From baseline to six months, there are no significant difference between the groups in the scales of part I (Table 2). The mean differences are generally positive which means that IBC has higher scores than DCIS. The confidence intervals are wide and indicate that IBC could have higher, lower, or the same scores as DCIS. At 18 months, there are a change in the trend where six out of nine scales showed significant differences between groups: IBC has significantly higher scores on the scales; Sense of dejection, Anxiety, Negative impact on behaviour, Breast examination, and Keeping my mind off things. The groups are not different in scores concerning Negative impact on sleep, Negative impact on sexuality, and Felt less attractive. At 36 months, there are generally no differences between the groups. Mean differences are positive on scale 1 to 5 and negative for 6 to 8 and all effect estimate have wide confidence intervals. At 14 years, we observe the same trends as for 36 months (Table 2).

**Table 2:** Adjusted and weighted analyses of COS-BC: Estimated mean differences, confidence intervals, and p-values. Higher scores indicate more psychosocial consequences. ^a^Only measured for women with breast cancer at 14 years. ^b^Only measured at 14 years.^c^ 0 is for “very poor” and 5 is “very good”.

	IBC compared to DCIS											
	Baseline		1 month		6 months		18 months		36 months		14 years	
	Mean Δ (95% CI)	p-value	Mean Δ (95% CI)	p-value	Mean Δ (95% CI)	p-value	Mean Δ (95% CI)	p-value	Mean Δ (95% CI)	p-value	Mean Δ (95% CI)	p-value
1. Sense of dejection (0–18)	1.33 (−0.85; 3.52)	0.23	1.65 (−0.71; 4.02)	0.17	2.11 (−0.94; 5.15)	0.18	2.38 (1.30; 2.94)	<0.001	1.34 (−0.26; 2.94)	0.10	1.31 (0.07; 2.54)	0.039
2. Anxiety (0–18)	2.26 (0.08; 4.44)	0.042	0.74 (−1.59; 3.02)	0.51	2.43 (0.20; 4.66)	0.033	2.36 (1.34; 3.38)	<0.001	1.17 (−0.41; 2.74)	0.15	1.04 (−0.10; 2.18)	0.07
3. Negative impact on behaviour (0–21)	2.16 (0.44; 3.88)	0.014	2.09 (−0.01; 4.19)	0.05	1.5 (−2.42; 5.42)	0.45	2.28 (1.11; 3.45)	<0.001	1.18 (−0.22; 2.59)	0.10	0.94 (−0.22; 2.10)	0.11
4. Negative impact on sleep (0–12)	1.17 (−0.22; 2.54)	0.10	0.33 (−1.45; 2.12)	0.72	1.02 (−1.19; 3.23)	0.37	1.17 (−0.56; 2.91)	0.19	0.49 (−0.63; 1.59)	0.39	0.63 (−0.29; 1.56)	0.18
5. Breast examination (0–6)	−0.12 (−1.03; 0.80)	0.80	0.33 (−0.46; 1.12)	0.45	0.69 (−0.05; 1.44)	0.07	1.02 (0.50; 1.53)	<0.001	0.87 (0.35; 1.38)	<0.001	−0.06 (−0.58; 0.46)	0.82
6. Negative impact on sexuality (0–6)	1.04 (−0.01; 2.10)	0.05	0.45 (−0.78; 1.68)	0.47	0.24 (−1.28; 1.77)	0.75	0.27 (−0.59; 1.37)	0.54	−1.04 (−2.23; 0.15)	0.09	−0.20 (−1.60; 1.19)	0.78
7. Felt less attractive (0–3)	−0.04 (−0.32; 0.23)	0.77	0.21 (−0.04; 0.45)	0.10	−0.08 (−0.44; 0.28)	0.66	−0.26 (−1.10; 0.57)	0.54	−0.30 (−0.75; 0.15)	0.20	−0.05 (−0.66; 0.57)	0.86
8. Keeping my mind off things (0–3)	−0.01 (−0.48; 0.47)	0.98	0.76 (−0.31; 0.66)	0.48	−0.27 (−1.17; 0.61)	0.54	0.60 (0.41; 0.81)	<0.001	−0.17 (−0.65; 0.31)	0.50	−0.04 (−0.62; 0.53)	0.88
9. Worries about breast cancer (0–4)^a^	–	–	–	–	–	–	–	–	–	–	0.78 (0.29; 1.26)	0.002
10. Inner calmness (0–6)	–	–	0.35 (−0.25; 0.94)	0.26	0.49 (−0.05; 1.03)	0.07	0.79 (0.29; 1.28)	0.002	0.32 (−0.30; 0.94)	0.31	0.98 (0.47; 1.05)	<0.001
11. Social network (0–6)	–	–	0.92 (0.29; 1.55)	0.005	0.81 (0.15; 1.46)	0.015	0.73 (0.23; 1.22)	0.004	0.55 (0.17; 0.94)	0.005	0.37 (0.05; 0.68)	0.020
12. Existential values (0–12)	–	–	1.37 (−0.07; 2.82)	0.07	−1.03 (−4.41; 2.34)	0.55	0.92 (−0.32; 2.17)	0.15	1.08 (−0.46; 2.62)	0.17	1.64 (0.75; 2.54)	<0.001
13. Impulsivity (0–12)^b^	–	–	–	–	–	–	–	–	–	–	0.64 (0.11; 1.17)	0.018
14. Empathy (0–6)^b^	–	–	–	–	–	–	–	–	–	–	0.82 (0.08; 1.54)	0.028
Self-rated health (0–5)^c^	–	–	−0.07 (−0.58; 0.43)	0.78	0.113 (−0.29; 0.55)	0.53	0.08 (−0.31; 0.47)	0.69	0.05 (−0.42; 0.53)	0.82	0.62 (0.22; 1.01)	0.002

Part II encompasses scales 9 to 14 (Table 2). At one month and six months, we observe generally positive mean differences meaning that IBC averagely higher scores thant DCIS. The confidence intervals are wide and indicate that IBC could have higher, lower, or the same scores as DCIS. At 18 months, women with IBC has significantly higher scores than DCIS on scales 10 and 11: Inner calmness and Social networks. For the Existential value scale 12, the mean difference is also positive but not significant. At 36 months, mean differences are positive and significant for the Social networks scale 11. At 14 years, all mean differences are positive with wide confidence intervals and the Inner calmness and Existential value scales 10 and 12 are significant.

For self-rated health, the mean differences were generally positive meaning here that IBC rated their health better than DCIS. From 1 month to 36 months, the confidence intervals are wide overlapping no difference between groups. At 14 years after diagnosis, IBC rated their health significantly better than DCIS.

## 4 Discussion

From baseline to six months after diagnosis, the groups experienced similar levels of psychosocial consequences. This indicates women with DCIS and IBC are equally psychosocially affected by their diagnosis. This might be due to the perceived threat of a cancer diagnosis. However, even though most mean differences were insignificant and had wide confidence intervals, the trend showed that IBC generally was more affected than DCIS. However, at 14 years, women with DCIS rated their overall health significantly worse than women with IBC. We also observed a trend that women with DCIS and IBC might be affected differently long term: mean scores and mean differences showed that IBC was more affected regarding the scale 1 to 5: Sense of Dejection, Anxiety, Negative impact on Behaviour, Negative impact on sleep, Breast examination, and in all six scales of Part II, and DCIS was more affected on the scale 6 to 8: Negative impact on sexuality, Felt less attractive, and Keeping my mind off things. Over time, the scores of both DCIS and IBC approached those of normal results.

### 4.1 Discussion of findings

The findings of this study are consistent with those of other studies [[19], [20], [21], [22], [23],^30^,^31^[32]]. These studies also suggest that women do not distinguish between DCIS and IBC, yet women diagnosed with DCIS overestimate their future risk of developing invasive breast cancer. A systematic review of the PROMs in DCIS found that across studies, women diagnosed with DCIS were equally affected concerning body image and sexuality as women with IBC [33].

Although DCIS and IBC have different prognoses, there is no evidence that the level of psychosocial consequences of being labelled with a potentially life-threatening cancer is any different. We know from previous research that women fear cancer and that cancer has an accessibility bias that frightens people and makes them unable to rationally weigh information about cancer [34]. If women do not distinguish the threat of IBC and DCIS, this means that these two groups are likely to require similar levels of psychosocial support. This study, however, shows that they might experience similar levels but that these are distributed differently. Providing adequate information about the prognosis and nature of DCIS may help to diminish the psychosocial consequences experienced by women diagnosed with DCIS. Previous studies found that women diagnosed with DCIS were confused, lacked knowledge about the condition and prognosis, and desired more information to support decisions [16,31,35,36]. A scoping review showed that in some studies, over half of women with DCIS were not aware that it was not invasive [16]. Providing adequate information about DCIS is especially important in screening programs where most cases of DCIS are detected [37].

For decades, research has advocated renaming DCIS to exclude the nomenclature of carcinoma. The cancer label of the low-risk tumor provides unnecessary psychological harm to patients, as well as influences women's decisions about management options towards a more invasive and aggressive treatment [[38], [39], [40][41]]. Therefore, renaming the low-risk cancer could potentially circumvent the extensive psychosocial impact of a DCIS diagnosis. An Australian interview study found that women preferred that the term did not include cancer and that excluding the nomenclature would enable discussions about and make them more comfortable choosing conservative management options [39[41]]. Researchers also argue that it might reduce confusion among health professionals and thereby reduce the risk of overtreatment [42]. Research has also shown that physicians prefer explanations and names for DCIS that exclude the nomenclature carcinoma [16,43]. This study emphasizes the continuous need for renaming DCIS. This has already been done for cervical intraepithelial neoplasia previously named cervical carcinoma in situ.

### 4.2 Strengths and limitations

Five surveys have previously conducted similar studies. However, they differ with respect to aim, follow-up time, and the questionnaire used [[19], [20], [21], [22], [23]]. The five surveys had findings similar to this study. This study supports these findings, but adds to the pile of evidence, because this study had a longer follow-up period than any of the five studies, had more measurement time points, used a questionnaire with adequate measurement properties, and used normal results as a benchmark. These are also the major strengths of this study. No study has previously followed women participating in screening for this long and therefore this study adds to the evidence about the long-term impact. Further, we use normal results as a benchmark to show that we are indeed able to reveal significant differences and as a benchmark for the magnitude of the psychosocial consequences. This study use the condition-specific questionnaire COS-BC; most similar studies use a battery of PROMs yet not developed or validated in a screening context. These may not be discriminative or sensitive enough to capture small impacts [44]. It is important to use a condition-specific questionnaire because any differences that may exist are likely to be too subtle for detection by generic measures.

Despite the strengths, this study has some limitations. First of all, the absolute number of women diagnosed with breast cancer was small and we therefore had low statistical power to detect differences. However, we did identify significant differences between the groups with p-values lower than 0.001. However, within the limits of our assumptions, the probability of a type I error is 1% for each statistical test. Thus, the findings of this study are in accordance with previous studies and prove biological plausibility that the first period after diagnosis is experienced equally burdensome; often referred to as the “the critical period”. Also, invasive treatment and more frequent hospital visits would expectedly affect women with IBC more than DCIS beyond the critical period. The fact that we were able to identify differences between the groups might be due to the sensitivity of the COS-BC. Another limitation is that the present study is a post-hoc analysis and thus results are exploratory.

The COS-BC has not established any thresholds for clinical important difference yet the structure of the questionnaire allows any score away from zero to be interpreted as a meaningful difference. Therefore, we focus on the overall pattern of differences. Further, this was out of the scope of this article, since we aimed to compare groups and not to question whether a cancer diagnosis has psychosocial consequences.

The study population is women who had participated in mammography screening. These findings might not be representative of diagnoses established outside of screening programs due to healthy worker bias. Potential attrition bias was accounted for with the method of IPW.

## 5 Conclusion

Renaming DCIS to exclude the nomenclature of cancer might decrease the psychosocial burden of diagnosis and help women make better decisions in accordance with the evidence, their values, and preferences. It might also support both health professionals and women in choosing more conservative management options and thereby avoiding overtreatment. Further, the finding that women with DCIS and IBC experience similar levels of psychosocial consequences long term, but in different ways could be explored in a study with larger sample size.

## Ethics approval and consent to participate

The study was approved by the Danish Data Protection Agency, 2007-41-0777. According to Danish Law on health research (§14, 2), questionnaire surveys do not require ethical approval. However, this study was conducted in accordance with the international guidelines stated in the Declaration of Helsinki and the participants gave informed consent.

## Funding

The Danish College of General Practitioners (DSAM) has financially supported the writing process of this research. The funding did not play any role in either parts of conducting, writing, or publishing this research.

## Declaration of competing interest

None declared.
